# A novel controllable capacitor commutation based superconducting hybrid direct current breaker

**DOI:** 10.1038/s41598-024-61129-9

**Published:** 2024-05-08

**Authors:** Yang Xu, Muhammad Junaid, Mingxue Li, Jinggang Yang, Yang Chen, Mohammed Alkahtani

**Affiliations:** 1 Electric Power Science Research Institute, State Grid Jiangsu Electric Power Co., Ltd., Nanjing, 211103 Jiangsu China; 2 School of Electrical Engineering, China University of Mining and Technology, Xuzhou, 221116 China; 3 Department of Electrical Engineering, College of Engineering, University of Bisha, P. O. Box 551, 61922 Bisha, Saudi Arabia

**Keywords:** Electrical and electronic engineering, Energy grids and networks

## Abstract

Featuring low power loss and high reliability, voltage source converter medium voltage direct current (VSC-MVDC) systems have been widely employed for grid-tied renewable energy applications. To maintain high operational safety, circuit breakers are needed to isolate faulted powerlines by comprehensively considering response speed and installation cost. Research efforts have been put to realizing DC fault isolation by coordinating resistive type superconducting fault current limiter (R-SFCL) and integrated-gate-commutated-thyristor (IGCT) based hybrid DC circuit breaker. In this paper, a controllable current commutation based superconducting DC circuit breaker (CCCB-SDCCB) is proposed. By integrating R-SFCL with IGCT based hybrid DC circuit breakers, the current interrupting capacity can be greatly enlarged with the advantage of low cost and fast speed, and hence the overall cost for suppress large fault currents can be greatly reduced for MVDC systems. In addition, a new current injection circuit branch using H-bridge structure is designed to recycle the residual capacitor voltage from the previous fault stage to trigger the IGCTs without the capacitor pre-charging process. Simulation results show that the fault current can be successfully suppressed from 24.2 to 2.1 kA and fully interrupted within 4.11 ms by the proposed CCCB-SDCCB.

## Introduction

With the rapid development of power electronics technology and renewable energy generation, the requirement of cooperatively controlling distributed renewable power generation, DC loads, and energy storage devices leads to great challenges to the AC power distribution networks^1^. The medium voltage direct current (MVDC) system has been widely adopted due to its low power loss, high reliability, and independent power control^2–4^. To control and protect the DC power transmission and distribution system, the MVDC circuit breaker is introduced as a safeguard to realize reliable and stable operation of the DC distribution system^5^. Generally, there are three main ways for interrupting the DC fault current in MVDC systems, namely AC circuit breakers, DC circuit breakers, and specific fault-blocking converter stations^6^. Due to the lack of current zero crossing in DC power systems, AC circuit breakers using zero crossings of the fault current to realize arc-less interruption cannot be utilized on the DC systems for fault current interruption. DC circuit breakers is one of the most promising solutions for isolating the faulty part of the MVDC and improving the system reliability. However, so far, most of the relevant researches on MVDC circuit breaker is still in the stage of theoretical explorations, and their penetration rate in engineering applications is still relatively low. In ref.^7^,  ± 10 kV solid-state DC circuit breaker based on insulated gate bipolar transistors (IGBTs) in serial connection was developed, a 5.1 kA short circuit current breaking test was carried out, and the dynamic and static equalization voltage of DC circuit breaker components was tested. In ref.^8^, the vacuum arc voltage characteristics of ± 10 kV medium-voltage DC circuit breaker were investigated, and a 3.6 kA/5 ms short circuit current breaking test was carried out. Considering the extremely high current rise rate di/dt under DC faults, fast isolation of the faulted line and fault current are quite crucial for the reliable operation of DC power systems^9–11^. A low voltage DC circuit breaker prototype has been built using a multistrand magnesium diboride (MgB2) coil, a vacuum interrupter, and an insulated-gate bipolar transistor module, which can realize interruption of 500 A DC within 4.4 ms^12^. A 1.5 kV DC circuit breaker involving a DC vacuum circuit breaker and a resistive-type SFCL in serial connection is discussed in ref.^13^, of which a DC vacuum circuit breaker is developed with a commutation circuit to generate a reverse injection current. The use of high-tech and economical DC circuit breakers to break fault currents is an effective solution for voltage source converter (VSC) MVDC multi-terminal DC system faults with point-to-point topology^14^.

The hybrid DC circuit breaker (HDCCB) combines excellent static characteristics of mechanical switches and outstanding dynamic performance of power electronic switches, which has the advantages of low conduction loss and fast switching speed^15,16^. Current commutating from the mechanical switch branch to the static current commutation branch is the precondition to successfully interrupt fault currents for the HDCCB. Hence, HDCCB also suffers from the difficulties of mechanical switch to extinguish arcs and the limited overload capacity of power electronic devices^17^. Therefore, considering these issues, resistive superconducting fault current limiters (R-SFCL) is introduced to the HDCCB to suppress the rising rate of fault current, thereby reduce the breaking pressure of the circuit breaker during DC grid faults^18,19^. In addition, through the rational design of R-SFCL ratings, the DC fault current and breaking current can be significantly reduced, and the safe and stable operation ability of high-voltage DC systems can be improved^20^. In ref.^21^, the cooperative characteristics of R-SFCLs and DC circuit breakers have been investigated with regard to the fault clearing in modular multilevel converter (MMC)-based multiterminal direct current (MTDC) grids. In ref.^22^, the performance of various circuit breaker topologies including ultrafast coupled inductor hybrid topology without and with the integration of R-SFCL is discussed for a 100 kV/100 MW HVDC transmission systems. It is found that the addition of R-SFCL with 1.7 ms response time decreases the peak current through the breaker as well as the power dissipated in the circuit breaker during short-circuit fault conditions. A protection method using a R-SFCL integrating with a solid-state DC circuit breaker to manage the DC short-circuit fault is proposed and experimentally verified in ref.^23^, where a bifilar SFCL coil prototype is designed to achieve low and high inductance to considerably reduce the fault current from 2000 A to below 1000 A. It is found that by integrating the SFCL with the solid-state DC circuit breaker, a high voltage is induced across the high inductance SFCL during current interruption tests. The commutation switches of main breaker utilizing IGBTs to commute and break fault current will increase the cost of power electronic components and HDCCBs^24,25^. Integrated-gate-commutated-thyristors (IGCTs) have the advantages of high rated current, fast commutation speed, high interference immunity, low cost, and high reliability, and thus have been widely used in DC power grids^26^. Therefore, as a much cheaper alternative, the IGCT-HDCCB can be applied in the medium voltage field as a main circuit breaker to break fault currents, which can considerably reduce the system cost^27^. However, for the current in 10 kV MVDC grid, the excessive fault current significantly increases the breaking pressure of the circuit breaker, and most capacitors in the existing circuit breaker structure require a pre-charging process. The additional pre-charging equipment and maintenance of capacitor voltage also increase the cost and control difficulty of the circuit breaker.

On this basis, a controllable capacitor commutation based superconducting DC circuit breaker (CCCB-SDCCB) is newly proposed in this paper. Unlike traditional circuit breakers, the proposed CCCB-SDCCB is a combination of R-SFCL and IGCT-based hybrid circuit breakers. The response time of R-SFCL is less than 1.2 ms, which can be used to quickly suppress the main branch current and achieve fast commutation after a very short-time circuit fault. In addition, the IGCT-based hybrid circuit breaker reduces the system cost while ensuring interrupting capability. The main circuit branch of CCCB-SDCCB consists of R-SFCL and an ultrafast disconnector switch (UDS), and the current commutation circuit is structured by IGCTs, thyristors, capacitors, and metal-oxide varistors (MOVs). As compared with the IGCT-HDCCB which requires pre-charging the capacitor before each short-circuit fault^28,29^, the bridge circuit composed of four thyristors can flexibly adjust the connection direction of the capacitor without setting up a separate pre-charging process. It fully utilizes the residual capacitor voltage from the previous fault stage to provide reverse shutdown voltage for the IGCTs in the next circuit breaking action, thereby ensuring the high current breaking capacity and low cost of the circuit breaker.

## R-SFCL model

**Figure 1:**
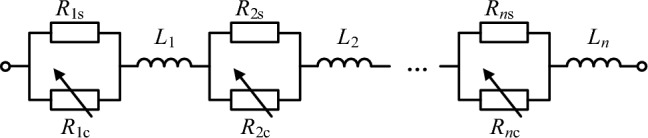
R-SFCL unit structure diagram.

The R-SFCL is utilized to automatically suppress the increase of short circuit current. Figure 1 shows the schematic structure of the R-SFCL unit, and the nth circuit unit consists of a resistor *R*_ns_, a superconducting resistor *R*_nc_, and a coil inductance *L*_n_, in which *R*_ns_ and *R*_nc_ are connected in parallel, and *L*_n_ is generally small enough to be negligible. When the circuit is operated in a steady state, the values of *R*_ns_ and *R*_nc_ are zero; when the circuit is undergoing an unexpected fault, the values of *R*_ns_ and *R*_nc_ will be increased abruptly. The total resistance of the R-SFCL depends on the total number of circuit units and the resistance of each unit, and hence the R-SFCL has zero resistance in the superconducting state and exhibits resistive characteristics in the quenching state^30,31^, which can limit the rapidly increasing fault currents and greatly reduce the shutdown pressure of the CCCB-SDCCB on high currents.

**Figure 2:**
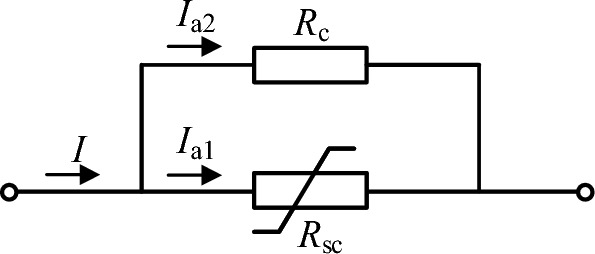
Simplified physical model of R-SFCL.

**Figure 3:**
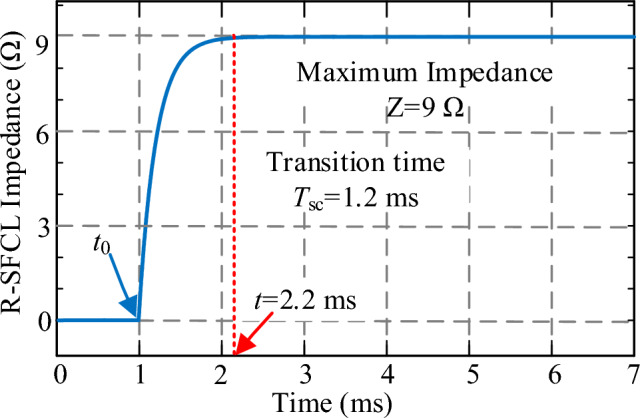
Quenching characteristics of R-SFCL.

The simplified physical model of R-SFCL is shown in Fig. 2. *R*_sc_ represents the current limiting resistance, which is zero in the superconducting state and can be greatly increased in the quenching state. *R*_c_ represents the bypass resistor that can prevent the superconductor from overcurrent. When R-SFCL is operated in the quenching state, its resistance value can be expressed as^32^:

1 $$ R_{{{\text{SFCL}}}} \left( t \right) = \left\{ \begin{aligned} & 0 \, t < t_{0} \\ & R_{{\text{m}}} \left( {1 - e^{{ - \frac{{t - t_{0} }}{{T_{{{\text{SC}}}} }}}} } \right) \, t > t_{0} \\ \end{aligned} \right., $$

where *t*_0_ is the onset time of quenching state, *T*_sc_ is the time constant of state transition, and *R*_m_ is the maximum resistance value of R-SFCL in the quenching state. Figure 3 shows the resistance curve of R-SFCL in the quenching state. When a short circuit fault occurs in the system at *t*_0_ = 1 ms, after a transition time of 1.2 ms, R-SFCL can reach the maximum resistance value. Therefore, it can be seen from Fig. 3 that the R-SFCL can effectively limit the rise of the DC fault current due to the rapid increase of R-SFCL resistance.

## Topology and operation principle of the proposed CCCB-SDCCB

**Figure 4:**
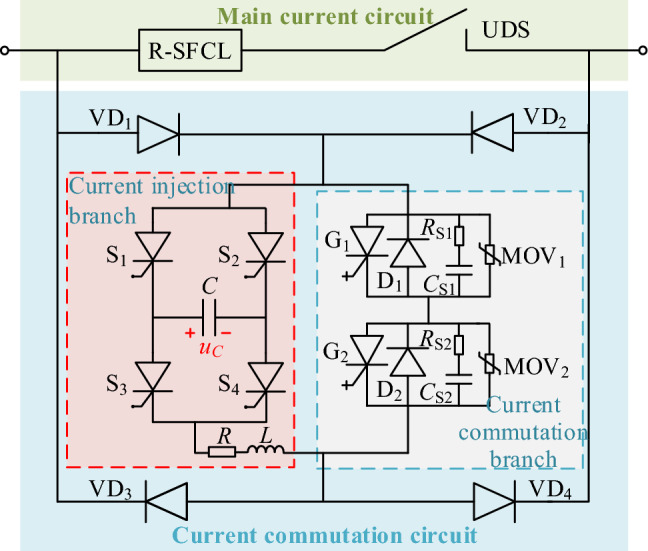
Topology of the proposed CCCB-SDCCB.

IGCT has great merits of high rated current, high commutation speed, low cost, and high reliability, etc., and it has been widely used in DC power systems. The proposed CCCB-SDCCB based on solid-state switching IGCT is shown in Fig. 4, of which the circuit main branch is composed of R-SFCL and UDS, and the current commutation circuit consists of four diodes VD_1_-VD_4_, the injection current branch, and the current commutation branch. Among them, R-SFCL is used to limit the fault current and achieve fast current commutation, and VD_1_-VD_4_ are used to provide circuit path for bidirectional current. The injection current branch consists of capacitor *C*, thyristor H-bridge (S_1_-S_4_), resistor *R*, and inductor *L*. This branch is used to rectify the fault current and provide shutdown voltage for the IGCTs, and hence the IGCT can be reliably shut down at small current levels. The current commutation branch is composed of two IGCTs, freewheeling diodes, snubber RCs, and MOVs connected in parallel.

**Figure 5:**
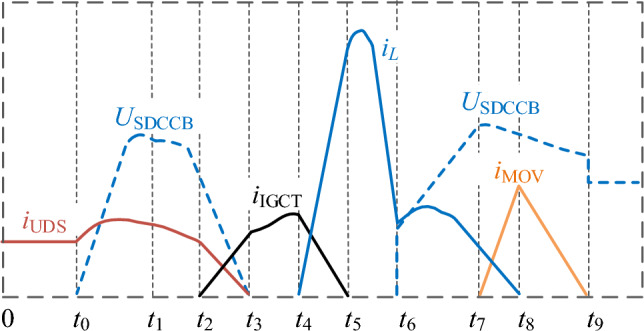
The typical current commutation and voltage waveforms during the whole interruption process.

According to the proposed topology shown in Fig. 4, the current commutation and voltage schematic of the proposed CCCB-SDCCB during the whole fault interruption process is shown in Fig. 5. Wherein, *i*_UDS_, *i*_IGCT_, *i*_L_, and *i*_MOV_ are the currents flowing through the UDS, IGCTs, the injection current branch, and the MOVs, respectively, while *U*_SDCCB_ is the voltage across the proposed CCCB-SDCCB. The normal operation mode as well as the whole fault interruption of the CCCB-SDCCB after a short-circuit fault is analyzed as follows.

When the powerline with SDCCB is in normal operation, i.e. *t* < *t*_0_, the load current flows through the powerline as well as the SDCCB. The steady state current *i*_0a_ of the system can be shown in Eq. (2), where *E* is the voltage of the DC side, *R*_load_ is the load resistance, and *Z*_1_ and *Z*_2_ are the powerline impedance.

2 $$ i_{{0{\text{a}}}} = \frac{E}{{R_{{{\text{load}}}} + Z_{1} + Z_{2} }}. $$

At time *t*_0_, a short-circuit fault is taking place in the system, the corresponding steady-state short-circuit current *i*_0b_ can be expressed by Eq. (3). Due to the high rising rate of the current, the R-SFCL automatically transforms from superconductive state to quenching state to reduce the rising rate of the fault current within about 1.2 ms. Then, after a controlled delay period, the UDS start to break the powerline.

3 $$ i_{{0{\text{b}}}} = \frac{E}{{Z_{1} + Z_{2} }}. $$

At time *t*_1_, a conduction signal is sent to activate the two IGCTs, G_1_ and G_2_. After a preset mechanical delay, at time *t*_2_, the current begins to commutate from the main current circuit to the current commutation circuit branch where G_1_ and G_2_ are located. The current then flows through diodes VD_1_, G_1_, G_2_, and diode VD_4_ in the current commutation circuit, until the current flowing through UDS drops to 0. This process is the first current commutation process after UDS action.

At time *t*_3_, the fault circuit commutation from the main current circuit to the current commutation circuit is completed.

At time *t*_4_, the UDS reaches its safe contact distance of circuit breaking, i.e., the current of the main current circuit drops to 0, then a corresponding pair of thyristors in the injection current branch are turned on according to the voltage polarity of the capacitor. When the capacitor voltage *u*_C_ > 0, S_2_ and S_3_ are turned on; when the capacitor voltage *u*_C_ < 0, S_1_ and S_4_ are turned on. By flexibly selecting and controlling the conduction of the thyristor pairs, the capacitor can provide reverse voltage for G_1_ and G_2_. Capacitor *C* is discharged via G_1_ and G_2_ so that these two IGCTs can be turned off with fast speed. Due to the connection of the injection current branch, the surge current is formed and passes through IGCTs G_1_ and G_2_, capacitor *C*, resistor *R*, inductor *L*, thyristors S_1_ and S_4_ (or S_2_ and S_3_). Then, the voltage of the capacitor can be decreased until 0. Meantime, the fault current is transferred from the current commutation branch to the injection current branch until it drops to 0. This is the second current commutation process after UDS action. The system fault current *i*_0c_ during this process satisfies

4 $$ \left\{ {\begin{array}{l} {E = L\frac{{{\text{d}}i_{{\text{L}}} }}{{{\text{d}}t}} + Ri_{{\text{L}}} + g_{{{\text{SCR}}}} u_{{\text{C}}} + 2u_{{{\text{diode}}}} + 2u_{{{\text{SCR}}}} } \\ {0 = L\frac{{{\text{d}}i_{{\text{L}}} }}{{{\text{d}}t}} + Ri_{{\text{L}}} + g_{{{\text{SCR}}}} u_{{\text{C}}} + 2u_{{{\text{SCR}}}} - 2u_{{{\text{IGCT}}}} } \\ {i_{{0{\text{c}}}} = i_{{\text{L}}} + i_{{{\text{IGCT}}}} } \\ \end{array} } \right., $$

5 $$ g_{{{\text{SCR}}}} = \left\{ {\begin{array}{*{20}c} 1 & {{\text{S}}_{1} \& {\text{S}}_{4} {\text{ are on}}} \\ { - 1} & {{\text{S}}_{2} \& {\text{S}}_{3} {\text{ are on}}} \\ \end{array} } \right., $$

where *u*_diode_ is the conduction voltage of freewheeling diodes VD_1_ and VD_4_, *u*_SCR_ is the conduction voltage of thyristors, *u*_C_ is the capacitor voltage, *i*_L_ is the injection current, and *i*_IGCT_ is the current of IGCTs.

At time *t*_5_, the two IGCTs G_1_ and G_2_ can be reliably turned off at a low current level, and the current is flowing through the parallel freewheeling diodes D_1_ and D_2_ of the IGCTs. At this moment, the current is flowing through diodes VD_1_ and VD_4_, *C*, *R*, *L*, S_1_, and S_4_ (or S_2_ and S_3_), and also forming a loop current in *C*, *R*, *L*, thyristors, freewheeling diodes D_1_ and D_2_. The system fault current *i*_0d_ during this process satisfies the following equations,

6 $$ \left\{ {\begin{array}{l} {E = L\frac{{{\text{d}}i_{{\text{L}}} }}{{{\text{d}}t}} + Ri_{{\text{L}}} + g_{{{\text{SCR}}}} u_{{\text{C}}} + 2u_{{{\text{diode}}}} + 2u_{{{\text{SCR}}}} } \\ {0 = L\frac{{{\text{d}}i_{{\text{L}}} }}{{{\text{d}}t}} + Ri_{{\text{L}}} + g_{{{\text{SCR}}}} u_{{\text{C}}} + 2u_{{{\text{SCR}}}} - 2u_{{\text{D}}} } \\ {i_{{0{\text{d}}}} = i_{{\text{L}}} + i_{{\text{D}}} } \\ \end{array} } \right., $$

where *u*_D_ is the conduction voltage of the freewheeling diodes, and *i*_D_ is the current of the freewheeling diodes.

At time *t*_6_, when the capacitor is discharged until its terminal voltage drops to 0, the capacitor will be charged in reverse by the fault current. The voltage across the current commutation circuit begins to rise in reverse, and the fault current is switched from the current commutation branch to the injection current branch. The second current commutation process ends. Thereafter, the current only flows through diodes VD_1_ and VD_4_, *C*,* R*, *L*, and thyristor pairs, and the system fault current *i*_0e_ in this process satisfies,

7 $$ \left\{ {\begin{array}{l} {E = L\frac{{{\text{d}}i_{{\text{L}}} }}{{{\text{d}}t}} + Ri_{{\text{L}}} + g_{{{\text{SCR}}}} u_{{\text{C}}} + 2u_{{{\text{diode}}}} + 2u_{{{\text{SCR}}}} } \\ {i_{{0{\text{e}}}} = i_{{\text{L}}} } \\ \end{array} } \right.. $$

At time *t*_7_, when the voltage at both ends of the current commutation circuit exceeds the conductive threshold of MOV_1_ and MOV_2_, the current switches from the injection current branch to MOV_1_ and MOV_2_, starting the third current reversal process;

At time *t*_8_, the thyristors S_1_ and S_4_ (or S_2_ and S_3_) are automatically turned off, and the current flows through diodes VD_1_ and VD_4_, surge arresters MOV_1_ and MOV_2_. The fault energy is depleted through MOVs. When the current rapidly drops below the minimum conductive threshold of MOV_1_ and MOV_2_, the lightning arrester returns to a high resistance state until the fault current reaches 0 at time *t*_9_. At this moment, the third current commutation process ends and the CCCB-SDCCB based on IGCT is disconnected. The energy consumption of the MOV in this process (i.e. *E*_MOV_) satisfies,

8 $$ E_{{{\text{MOV}}}} = \int_{{t_{7} }}^{{t_{9} }} {U_{{{\text{MOV}}}} i_{{{\text{MOV}}}} {\text{d}}t} = {{U_{{{\text{MOV}}}} I_{{{\text{peak}}}}^{2} } \mathord{\left/ {\vphantom {{U_{{{\text{MOV}}}} I_{{{\text{peak}}}}^{2} } {2\left( {\frac{{{\text{d}}i_{{{\text{MOV}}}} }}{{{\text{d}}t}}} \right)_{{{\text{avg}}}} }}} \right. \kern-0pt} {2\left( {\frac{{{\text{d}}i_{{{\text{MOV}}}} }}{{{\text{d}}t}}} \right)_{{{\text{avg}}}} }}, $$

where *t*_7_ and *t*_9_ represent the moments when the MOVs begin to consume fault energy and the fault current drops to 0, respectively. *U*_MOV_ is the threshold voltage of the MOV, *i*_MOV_ is the MOV current, *I*_eak_ is the peak value of the MOV current, and (d*i*_MOV_/d*t*)_avg_ is the average rate of fault current decrease in the MOV.

In addition, the fault clearance time Δ*t* is,

9 $$ \Delta t = T_{{\text{d}}} + {{i_{{{\text{peak}}}} } \mathord{\left/ {\vphantom {{i_{{{\text{peak}}}} } {\left( {\frac{{{\text{d}}i_{{{\text{MOV}}}} }}{{{\text{d}}t}}} \right)_{{{\text{avg}}}} }}} \right. \kern-0pt} {\left( {\frac{{{\text{d}}i_{{{\text{MOV}}}} }}{{{\text{d}}t}}} \right)_{{{\text{avg}}}} }}, $$

where *T*_d_ is the shutdown delay time of the IGCT.

**Figure 6:**
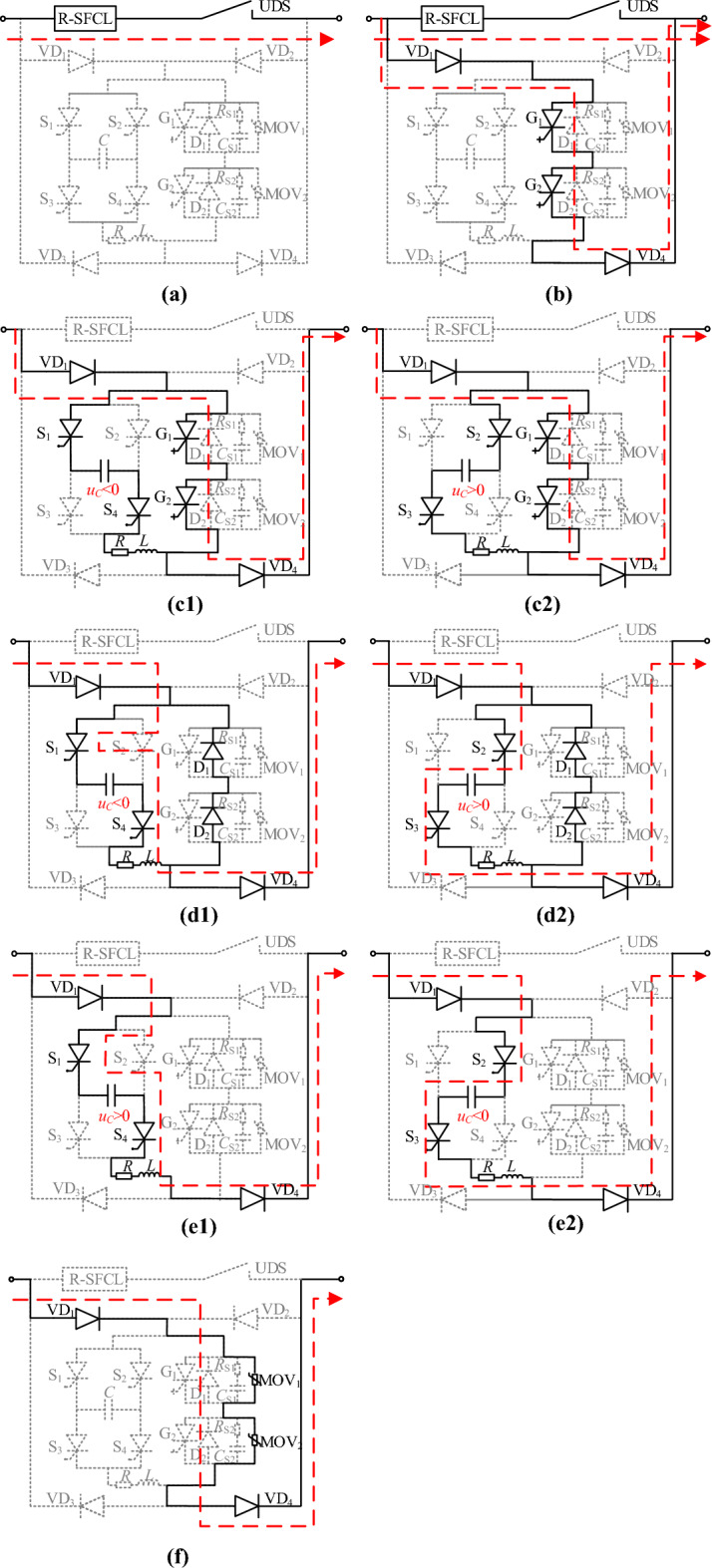
The current commutation process in the CCCB-SDCCB during different fault current interruption periods.

The circuit paths of fault current during the operation of CCCB-SDCCB in the above processes are shown in Fig. 6. It can be seen from Fig. 6a, when a short-circuit fault occurs in the MVDC system, the R-SFCL is automatically switched to superconductivity quenching state with nonzero resistance to suppress the fault current to a lower level. Then, UDS starts to operate with limited fault current. Figure 6b shows the flowing path of the commutation current from the main circuit branch to the current commutation branch during the UDS tripping process. As shown in Fig. 6c, when the thyristors S_1_ and S_4_ are conducted, capacitor *C* is discharged to the current commutation circuit branch and the fault current is commutated from the current commutation branch to the current injection branch. Figure 6d reveals that after the IGCT is turned off, the discharging current of the capacitor is flowing through the reverse parallel diodes of the commutation circuit branch. Figure 6e shows that as the fault current is flowing through the current injection branch, capacitor C is charged in reverse, allowing to be utilized in the next fault process, and the voltage across the proposed CCCB-SDCCB begins to rise. Figure 6f indicates that after the voltage across the proposed CCCB-SDCCB crosses the arrester threshold, the fault current is routed through the arrester to discharge the fault energy, and the fault current drops rapidly until reaching zero, and the fault stage ends.

## Simulation and discussion

**Figure 7:**
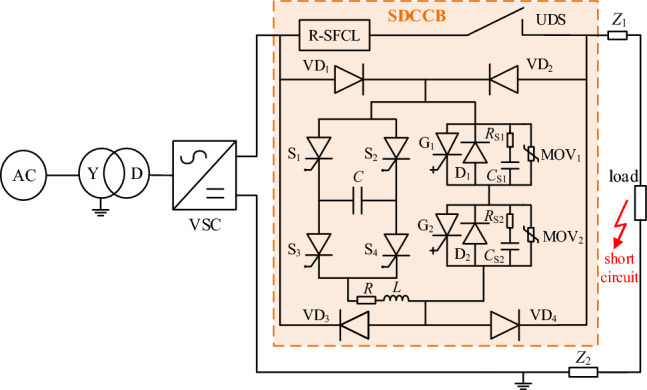
Simulation schematic of the proposed topology structure.

To verify the proposed CCCB-SDCCB in this paper, a 10 kV VSC-MVDC system simulation model was built by MATLAB/Simulink, and the diagram of system structure is shown in Fig. 7.

The output voltage of the rectifier is 10 kV, the load resistance is 10 Ω, the rated current is 1 kA, and the rated power is 1 MW. When a short-circuit fault occurs in the load side, the short circuit resistance is 0.1 Ω, and the CCCB-SDCCB performs to limit the fault current and break the short-circuit powerline. In the CCCB-SDCCB, the maximum resistance of R-SFCL is 9 Ω, which is the key to limit the fault current to 2.1 kA. The RC buffer branch is structured by a 10 μF capacitor and a 5 Ω resistor, with a rated voltage of 6.5 kV for MOV. The capacitor of the current injection branch is set to 0.3 mF, with a voltage of 14.3 kV. The resistance and inductance are 0.4 Ω and 7 μH, respectively. The following is a validation of the interrupt performance of CCCB-SDCCB and the operation performance of R-SFCL in CCCB-SDCCB.

### Fault interruption performance of CCCB-SDCCB

**Figure 8:**
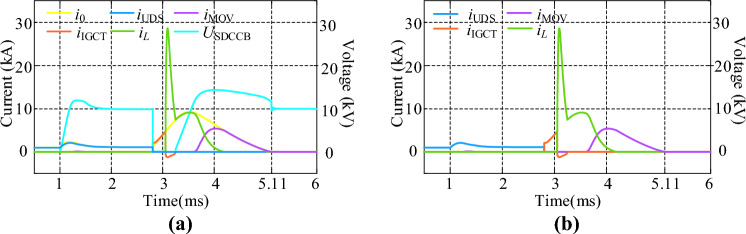
Main process of CCCB-SDCCB Operation under Fault. (**a**) Overall waveform. (**b**) Detailed commutation waveform.

The main waveforms of CCCB-SDCCB under a short circuit fault in a 10 kV MVDC system is shown in Fig. 8, where *i*_0_ is the total current of the powerline. As shown in Fig. 8a, when the fault occurs at *t* = 1 ms, the fault current is limited from 24.2 kA to 2.1 kA within 1.2 ms through the current limiting effect of R-SFCL quenching state, and then the UDS of the main circuit branch begins to break the powerline. The conduction signal is sent to turn on G_1_ and G_2_, and after a preset mechanical delay, the fault current is switched to the current commutation circuit branch after a delay of 3 μs. During this time interval, the voltages at the two ends of the *U*_SDCCB_ are equal due to its parallel connection with the R-SFCL. When the two contacts of UDS reach the safe breaking distance, assuming that the capacitor voltage *u*_C_ > 0, a conduction signal is sent to turn on the thyristors S_1_ and S_4_ (otherwise turn on the thyristors S_2_ and S_3_), and capacitor C is discharged to the current commutation circuit branch and forces IGCTs to be turned off. When *t* = 3.05 ms, the fault current is commutated from the current commutation branch to the current injection branch and this commutation process ends at *t* = 3.25 ms. Due to the fault current reversing from the IGCT to the current injection branch during this process, the capacitor is discharged so that its voltage decreases to 0. Then, the fault current start to charge capacitor C in reverse, resulting in the capacitor voltage rising in the opposite polarity. When the voltage of the capacitor exceeds the preset threshold voltage of the MOVs, energy stored in capacitor is released through the MOVs. The MOV starts consuming the fault energy of the system at approximately *t* = 3.55 ms. When *t* = 5.11 ms, the current reaches 0, indicating that the fault is completely cleared. In summary, the interruption process lasts for a total time of 4.11 ms.

Therefore, the proposed CCCB-SDCCB in this paper has a fault current breaking capacity of 10 kV/24.2 kA, which can break the fault current within 4.11 ms. The fault current breaking capacity is also determined by the rated parameters of the devices, such as the R-SFCL, the thyristors, and diodes. The Fig. 8b shows the detailed current commutation process during the CCCB-SDCCB operation. As analyzed in the previous text, the current commutation occurs for three times in total during the whole circuit breaker operating process under fault. The first current commutation is realized based on the conduction of the IGCTs, and the current is commutated from the main current loop to the current commutation branch. The second current commutation is triggered by the voltage on capacitor C to force the IGCTs turn off, and the current is switched from the current commutation branch to the current injection branch. The third current commutation is triggered by the capacitor voltage exceeding the MOV threshold, causing the current to flow from the current injection branch to the current commutation branch of the MOV, and up to this point the fault energy is released through the MOVs until fully depleted.

### R-SFCL behavior in CCCB-SDCCB

**Figure 9:**
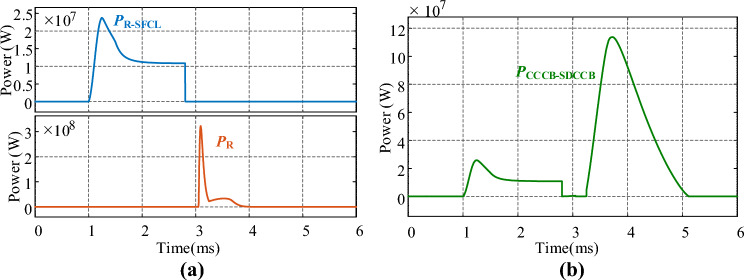
Power absorbing by (**a**) R-SFCL and resistor *R*. (**b**) The proposed CCCB-SDCCB.

R‐SFCL is a significant device for the proposed CCCB-SDCCB because it can suppress the rising rate of short-circuit current and reduce the pressure on the CCCB-SDCCB interrupting fault current. For normal operation conditions, the R-SFCL is operated at superconducting state with zero resistance. Hence, the power loss caused by the resistance of the R-SFCL in normal operation cases can be ignored. For operation conditions with fault current, the current passing through R-SFCL is greatly grown to a very high value, and hence the current density of R-SFCL is also greatly increased so that exceed the critical values. Therefore, the R-SFCL will be automatically transitioned from superconducting state to a superconductivity quenching state by the increased current density. In this case, the resistance of the R-SFCL is no longer zero and rapidly increased to suppress the fault current. Hence, the power loss caused by the R-SFCL under fault operation conditions is no longer zero. As shown in Fig. 9a, the power loss of the R-SFCL resistance can be rapidly increased due to the high fault current in a very short time. Then, the current injection branch and the current commutation branch are activated and the power loss of resistor R inside the current injection branch is also shown in Fig. 9a. The power loss curve of the proposed CCCB-SDCCB is shown in Fig. 9b. It can be seen that during normal operation, the power loss of the proposed CCCB-SDCCB is almost zero; for fault operation conditions, the power loss is mainly caused by the R-SFCL, the charging resistor R and the MOVs. However, the R-SFCL as well as the resistor R and MOVs are operating together to consume/absorb the fault power, which is conducive to the breaking operation of the proposed CCCB-SDCCB, and hence will not affect the power transmission efficiency.

**Figure 10:**
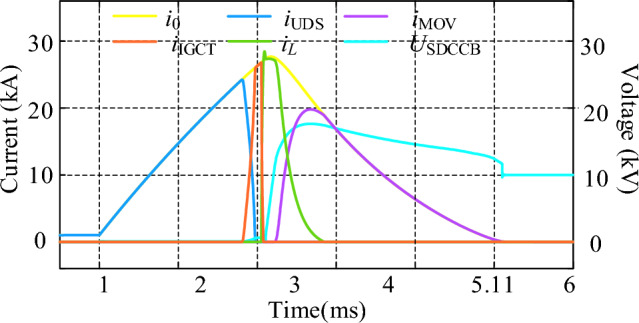
Overall waveforms of CCCB-SDCCB under fault without R-SFCL.

To compare the current suppression effect of R-SFCL, the overall waveforms of the proposed CCCB-SDCCB operated under faults without R-SFCL is shown in Fig. 10. It can be seen that, without R-SFCL, the short-circuit current can reach up to 24.2 kA. The entire operation process of the CCCB-SDCCB without R-SFCL can be analyzed by referring Fig. 10. The fault current is converted from the UDS to the two IGCTs for 0.18 ms, and the current going through IGCTs can reach up to 24.8 kA. When the thyristors S_1_ and S_4_ of the current injection branch are turned on, the current of IGCTs gradually decreases by the reverse charging of the capacitor at *t* = 3.05 ms. Until *t* = 3.08 ms, the current in these IGCTs drops to 0 and they can be turned off, causing the fault current commutating to the current injection circuit branch and charging the capacitor. The maximum current of the thyristors is 28.45 kA, and the voltage of the capacitor rises reversely. The MOV starts to dissipate the fault energy of the system at about *t* = 3.23 ms with the maximum current of 19.8 kA, and the whole interruption process lasts for a total of 5.1 ms. Thus, Figs. 8 and 10 demonstrate that, the proposed CCCB-SDCCB with R-SFCL has a much smaller fault current and a shorter fault clearing time.

**Figure 11:**
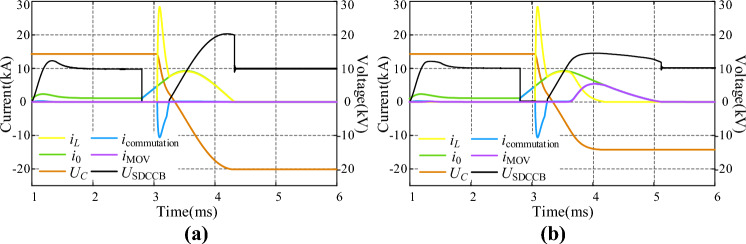
Current and voltage with and without current absorbing. (**a**) With current absorbing. (**b**) Without current absorbing.

Lightning arrester MOV is a reliable energy absorbing and voltage limiting element, and the commonly used oxide lightning arrester is zinc oxide (ZnO) lightning arrester. The fault current going through MOV can rapidly reach thousands of amperes. Without MOV, the fault energy remained in equivalent system inductors and capacitors will cause very high voltage or current stress on the proposed CCCB-SDCCB. In addition, the reverse charging time of capacitor *C* in the injection current branch will be enlarged, and the reverse charging voltage and current of capacitor *C* will be greatly increased, which is not conducive to improving the efficiency and service life of the commutation capacitor. The waveforms of current and voltage of the proposed CCCB-SDCCB with and without energy absorbing MOV are shown in Fig. 11, where the current going through MOV is represented by the purple curve. It can be observed that without MOV, the peak value of voltage *U*_SDCCB_ and *U*_C_ can be greatly reduced.

**Figure 12:**
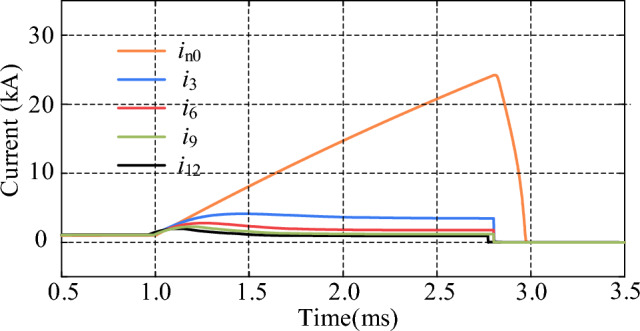
Fault current in the main branch for changing values of *Z*_R-SFCL_.

**Figure 13:**
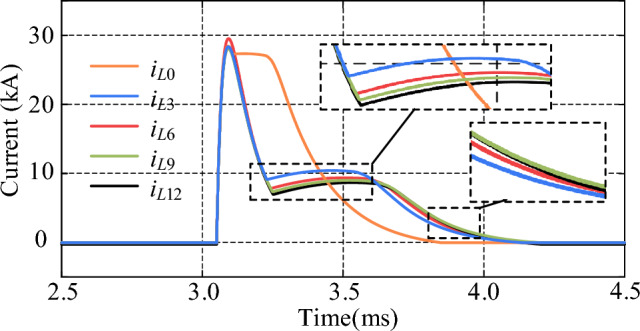
Fault current of thyristor for changing values of *Z*_R-SFCL_.

By comparing the cases of using R-SFCLs connected with and without *Z*_R-SFCL_ in the main circuit branch, its effect on the main circuit branch and thyristor fault currents can be observed and compared. As shown in Fig. 12, *i*_n0_ represents the current in the main branch when the *Z*_R-SFCL_ is 0 Ω, and *i*_3_, *i*_6_, *i*_9_, and *i*_12_ represent the currents flowing through the main branch when the *Z*_R-SFCL_ is 3 Ω, 6 Ω, 9 Ω, and 12 Ω, respectively. When* Z*_R-SFCL_ = 0 Ω, the fault current can reach up to 24.2 kA, which means there is a high requirement for the shutdown capability of the CCCB-SDCCB. When *Z*_R-SFCL_ = 3 Ω, the fault current can be decreased with the maximum value of 3.85 kA, and the time required to turn off the fault current is also decreased. The fault current can be decreased by increasing *Z*_R-SFCL_, which indicates that larger *Z*_R-SFCL_ can lead to lower rising rate of fault current in the MVDC system, as well as smaller shutdown pressure of the CCCB-SDCCB. The current flowing through UDS and the percentage of fault current reduction by changing the *Z*_R-SFCL_ value are shown in Table 1. The comparison results of the fault current of thyristors when the *Z*_R-SFCL_ value is changed are shown in Fig. 13. The results show that when the *Z*_R-SFCL_ is configured to 0 Ω, 3 Ω, 6 Ω, 9 Ω, and 12 Ω, the maximum current values of thyristors are 28.45 kA, 28.37 kA, 29.54 kA, 28.41 kA, and 28.63 kA, respectively. Therefore, a reasonable selection of the maximum R-SFCL resistance value can reduce the current stress of thyristors.

**Table 1:** Percentage reduction of main branch fault current for changing *Z*_R‐SFCL_.

*Z*_R-SFCL_	Current	Percentage reduction
0 Ω	24.2 kA	–
3 Ω	3.85 kA	84.1%
6 Ω	2.60 kA	89.3%
9 Ω	2.10 kA	91.3%
12 Ω	1.81 kA	92.5%

**Figure 14:**
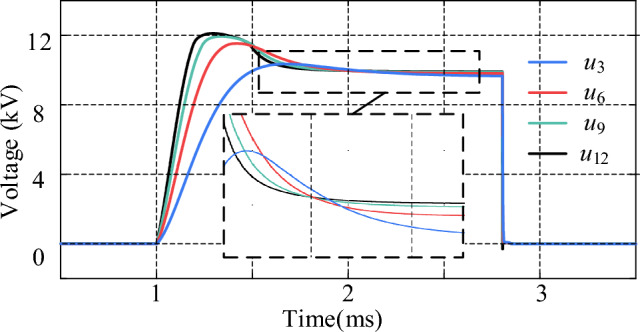
Voltage across R-SFCL for changing values of *Z*_R-SFCL_.

The measured voltages across the R-SFCL (*V*_R-SFCL_) under short-circuit faults in the MVDC system are shown in Fig. 14, where *u*_3_, *u*_6_, *u*_9_, and *u*_12_ represent the voltages across the R-SFCL when the *Z*_R-SFCL_ is configured to 3 Ω, 6 Ω, 9 Ω, and 12 Ω, respectively. It can be found that the voltage across R-SFCL approaches the maximum value when *Z*_R-SFCL_ = 12 Ω; As *Z*_R-SFCL_ is decreased, the voltage across R-SFCL can be gradually decreased, which means lower R-SFCL resistance leads to lower rated voltage. Hence, high R-SFCL resistance is conducive to reduce the breaking current pressure of the proposed CCCB-SDCCB. According to Figs. 12 and 14, the parameters of R-SFCL has different effect on its rated voltage as well as turn-off current capability. However, when the quenching resistance of the R-SFCL is large, the large resistance will result in high rated voltage requirements, as well as the overall cost of the R-SFCL. Therefore, it is necessary to design the R-SFCL by making rounds of these two indicators according to the actual application requirements. In summary, *Z*_R-SFCL_ = 9 Ω is selected in this paper to achieve the maximum cost-effectiveness between small fault current and cost.

**Figure 15:**
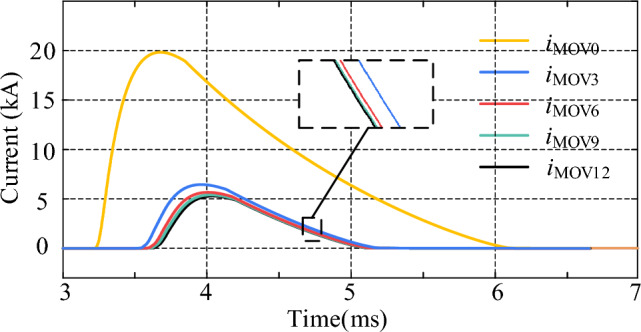
Current flowing through MOV for changing values of *Z*_R-SFCL_.

The waveforms of the current flowing through the MOV at different *Z*_R-SFCL_ values are shown in Fig. 15. It can be seen that the current flowing through the MOV can be significantly decreased by increasing *Z*_R-SFCL_, and the rate of its rising current also decreases. In addition, the transition time of MOV current at *Z*_R-SFCL_ = 0 Ω is longer than that of *Z*_R-SFCL_ values. This indicates that the fault clearing time can be shortened by increasing the *Z*_R-SFCL_ value, and thus the R-SFCL also contributes to absorbing the residual fault energy.

### The influence of circuit parameters on the current of thyristors

**Figure 16:**
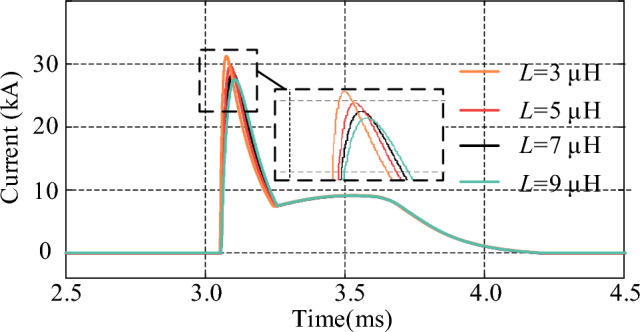
Thyristor current for different values of *L*.

**Figure 17:**
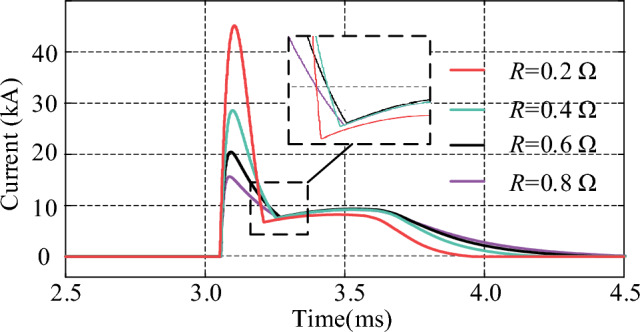
Thyristor current for different values of *R*.

**Figure 18:**
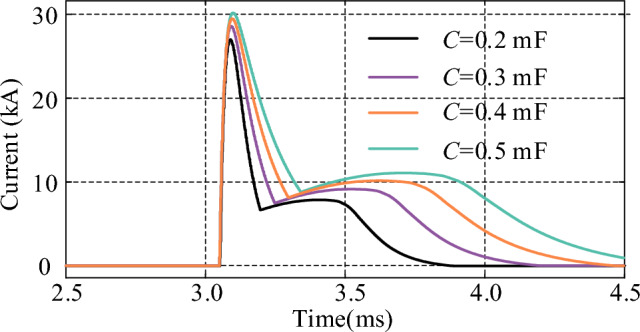
Thyristor current for different values of *C*.

The circuit parameters of the injection current branch have a decisive impact on the interruption capability of the proposed CCCB-SDCCB. In this section, the thyristor currents are simulated and analyzed under different resistances, inductances, and capacitances by tuning the circuit parameters, to evaluate the impact of different parameter value on the interrupt capability of the CCCB-SDCCB. Figures 16, 17 and 18 show the simulation waveforms of the thyristor currents under different inductance, resistance, and capacitance values. It can be seen that the thyristor current decreases as the inductance or resistance of the injection current branch increases, which means a low capability of breaking the fault current for the CCCB-SDCCB. On the contrary, the thyristor current increases as the capacitance of the injection current branch increases. Thyristors with higher rated current could endow the circuit breaker higher capability to break the fault current. Therefore, the breaking capability of the CCCB-SDCCB can be improved by decreasing the inductance to increase the current commutation frequency under the condition of constant capacitance. Alternatively, the capacitance can be appropriately increased to improve the reliability of the breaking operation of fault current under the premise of guaranteeing good insulating characteristics of the capacitor.

In summary, the capability of breaking the fault current for the CCCB-SDCCB should be greater than 25 kA in a 10 kV/24.2 kA MVDC system. Considering the volume and cost constraints, the parameters of the current injection branch components are therefore selected as *L* = 7 µH, *R* = 0.4 Ω, and *C* = 300 µF.

## Conclusions

A controllable CCCB-SDCCB for isolating short-circuit faults in 10 kV MVDC systems is proposed in this paper. Firstly, a mathematical model of the R-SFCL is established to analyze the resistance characteristics. Then, a CCCB-SDCCB for 10 kV/24.2 kA MVDC is proposed and the voltage-current characteristics are analyzed under each operation stage. The circuit breaker adopts a R-SFCL to limit the short-circuit current rising rate and absorb residual fault energy. It is coupled with IGCTs to ensure the fault current suppression and reliable current commutation for the CCCB-SDCCB to break the fault current, which can reduce the hardware cost of the circuit breaker. In addition, the current injection branch with a controllable commutation structure fully utilizes the residual capacitor voltage from the previous fault stage. It can accelerate the turn-off process of the IGCTs, without the process of pre-charging the capacitor before each fault. The proposed CCCB-SDCCB can achieve reliable and fast disconnection of short circuit powerlines in 10 kV MVDC systems. The proposed current injection and commutation circuit branch endow the proposed CCCB-SDCCB also with low cost and long service life. A simulation platform was built to comparatively test the breaking performance of CCCB-SDCCB in terms of different parameters. The simulation results demonstrate that by configuring the quenching resistance of R-SFCL to 9 Ω, the proposed CCCB-SDCCB can limit the fault current from 24.2 kA to 2.1 kA (about 91.3%) and achieve current breaking within 4.11 ms.

## Data Availability

The datasets used and/or analyzed during the current study are available from the corresponding author on reasonable request.
